# Terminal Digit Preference and Threshold Avoidance in Digital Blood Pressure Measurements During Pregnancy: Secondary Analysis of Data From the CLIP and PRECISE Cohorts

**DOI:** 10.2196/73307

**Published:** 2026-06-03

**Authors:** Peter von Dadelszen, Akshdeep Sandhu, Jeffrey N Bone, Rahat N Qureshi, Olukayode A Dada, Ashalata A Mallapur, Marleen Temmerman, Marie-Laure Volvert, Joseph Waiswa, Moses Mukhanya, Hiten D Mistry, Shivaprasad S Goudar, Hawanatu Jah, Angela Koech, Charfudin Sacoor, Anifa Vala, Khatia Munguambe, Anna Roca, Olelekan O Adetoro, Esperanca Sevene, Marianne Vidler, Mrutyunjaya B Bellad, John Sotunsa, Hannah J Blencowe, Zulfiqar A Bhutta, Umberto d'Alessandro, John Mark Ansermino, Guy A Dumont, Laura A Magee, CLIP Trials Study Group, Esperança Sevene, PRECISE Network, Umberto D'Alessandro

**Affiliations:** 1 Department of Women and Children’s Health, School of Life Course and Population Sciences, Faculty of Life Sciences and Medicine, King's College London, 6th Floor, Addison House, Guy's Campus, Great Maze Pond, London, SE1 1UL, United Kingdom, 44 7484163625; 2 Department of Obstetrics and Gynaecology, University of British Columbia, Vancouver, BC, Canada; 3 BC Children's Hospital Research Institute, Vancouver, BC, Canada; 4 Centre of Excellence for Women & Child Health, Aga Khan University, Karachi, Sindh, Pakistan; 5 Centre for Research in Reproductive Health, Sagamu, Ogun State, Nigeria; 6 S Nijalingappa Medical College and HSK Hospital & Research Centre, Bagalkot, Karnataka, India; 7 Centre of Excellence in Women and Child Health (East Africa), Aga Khan University Nairobi, Nairobi, Kenya; 8 MARCH Centre, London School of Hygiene & Tropical Medicine, London, United Kingdom; 9 Women's and Children's Health Research Unit, KLE Academy of Higher Education and Research, Jawaharlal Nehru Medical College, Belagavi, India; 10 MRC Unit The Gambia at the London School of Hygiene and Tropical Medicine, Banjul, Fajara, The Gambia; 11 Centro de Investigação em Saúde da Manhiça, Manhiça and Department of Clinical Pharmacology, Faculdade de Medicina, Universidade Eduardo Mondlane, Manhiça, Mozambique; 12 Department of Obstetrics and Gynaecology, Babcock University Teaching Hospital, Ilishan-Remo, Ogun State, Nigeria; 13 Centre for Global Child Health, Hospital for Sick Children, Toronto, ON, Canada; 14 Department of Electrical and Computer Engineering, University of British Columbia, Vancouver, BC, Canada; 15 See Acknowledgments

**Keywords:** Africa, blood pressure devices, pregnancy, South Asia, terminal digit preference, threshold avoidance, women of reproductive age

## Abstract

**Background:**

Screening for, detecting, and managing pregnancy hypertension is a core function of antenatal care. To reduce both training requirements and the risks of measurement error in blood pressure (BP) values, automated and semiautomated BP devices have been validated in pregnant women with normal BP and pregnant women with hypertension and introduced for serial antenatal measurement of BP.

**Objectives:**

The study aimed to (1) determine whether or not repeated BP measurements reduced the presence of terminal digit preference and (2) discern whether or not there was evidence of threshold avoidance in the Community-Level Interventions for Preeclampsia (CLIP) trials compared with the purely observational Pregnancy Care Integrating Translational Science, Everywhere (PRECISE) cohorts.

**Methods:**

The BP 3AS1-2 and CRADLE Vital Signs Alert low-cost Microlife BP devices were used by trained research staff in the CLIP trials conducted in India, Mozambique, Nigeria (pilot trial only), and Pakistan and the PRECISE cohorts of unselected pregnant women and nonpregnant women of reproductive age recruited in the Gambia, Kenya, and Mozambique. Both devices algorithmically calculate systolic blood pressure and diastolic blood pressure values displayed on digital read-outs. All BP readings were entered manually into a digital platform, which averaged them as the BP for that visit; the first and second readings were averaged unless they were more than 10 mm Hg different, which triggered a third reading, and the second and third readings were averaged.

**Results:**

A total of 51,875 participants had their BP measured 438,404 times. Using raw BP values, there was terminal digit preference (129,539/911,500, 14.21% vs 10%; *P*<.001 values ended in zero). A total of 28,929 out of 437,446 (6.61%) dBP values were 62 mm Hg, compared with 9310 of 195,349 (4.77%) from the averaged values (*P*<.001); errors were obviated by averaging BP values. There was evidence of both threshold preference and avoidance in the CLIP trials and the PRECISE cohort.

**Conclusions:**

Given the excess of 62 mm Hg values, there is a shared inherent algorithmic error in the calculation of dBP in the BP 3AS1-2 and CRADLE Vital Signs Alert devices. Averaged BP measurements are important to reduce the impact of user errors in manually recording BP values. We recommend that automated and semiautomated BP devices should be connected wirelessly to automatically transfer readings to digital health records to further optimize care.

## Introduction

Pregnancy hypertension (defined as systolic blood pressure [sBP] of at least 140 mm Hg or diastolic blood pressure [dBP] of at least 90 mm Hg) is associated with approximately 46,000 maternal and 500,000 perinatal deaths annually; more than 99% of those deaths occur in low-income and middle-income countries [1,2]. Pregnancy hypertension is classified as chronic (pre-existing) hypertension, gestational hypertension, and preeclampsia [2]. Screening for, detecting, and managing pregnancy hypertension is a core function of antenatal care [2,3].

To improve access to BP and other vital signs measurement in low-income and middle-income countries, the semiautomated, low-cost Microlife BP 3AS1-2 and CRADLE Vital Signs Alert (VSA) devices were developed and have been validated in pregnant women with normal BP and pregnant women with hypertension [4,5]. In the context of the CLIP (Community-Level Interventions for Preeclampsia) trials, per protocol use of the Microlife BP 3AS1-2 device within a digital health package deployed by community health workers conducting antenatal contacts in women’s homes cost-effectively improved maternal and perinatal survival [6-11].

There are a number of caveats when measuring BP. First, individuals should rest for at least 5 minutes, and their left arm should be supported, with the cuff at the level of their heart. Second, at least 2 measurements of BP should be taken, and if there is discordance between the first and second reading, at least 1 additional measurement should be taken, with the final 2 values of both sBP and dBP being averaged [12]. BP readings that are not wirelessly connected to a digital application are susceptible to a number of errors, such as terminal digit preference (ie, disproportionately enumerating numbers that end in “0” or “5”) and threshold avoidance (ie, enumerating numbers just below the threshold that mandates a clinical response) [13].

In this study, we studied all the sBP and dBP values of the pregnant women who participated in the CLIP trials (including the Nigerian pilot trial) and the nonpregnant women of reproductive age and pregnant women who participated in the PRECISE (Pregnancy Care Integrating Translational Science, Everywhere) network. We aimed to (1) determine whether or not repeated BP measurements reduced the presence of terminal digit preference; and (2) discern whether or not there was evidence of threshold avoidance in the CLIP trials compared with the purely observational PRECISE cohort.

## Methods

### Study Design

This is a secondary analysis of data collected in the 4 countries and 27 intervention clusters of the CLIP cluster randomized controlled trials [14], in India (Karnataka: n=6) [6], Pakistan (Sindh Province: n=10) [7], Mozambique (Maputo and Gaza Provinces: n=6) [8], and Nigeria (Ogun State: n=5) [9], and from all participants in the 3-country PRECISE Network [15], in Gambia (n=3 primary health centers, Farafenni District), Kenya (n=2 primary health centers, Kilifi County), and Mozambique (n=2 primary health centers, Maputo Province; Table S2 in Multimedia Appendix 1). A STROBE (Strengthening the Reporting of Observational Studies in Epidemiology) checklist is provided (Checklist 1).

### The CLIP Trials

In brief, pregnant women (aged 15‐49 y in India, Pakistan, and Nigeria; and 12‐49 y in Mozambique) were enrolled in the CLIP trials when they first declared their pregnancy and following informed consent [6-9]. The CLIP intervention consisted of community engagement and community health worker-provided mobile health-guided clinical assessment, initial treatment, and referral to facility [14].

The CLIP intervention was implemented in primarily rural areas of India (February 2014-October 2016), Pakistan (February 2014-December 2016), Nigeria (March 2014-January 2016), and Mozambique (February 2015-February 2017). Within each country, clusters consisted of an established unit of the health system (ie, primary health center in India, union council in Pakistan, administrative post in Mozambique, and local government area in Nigeria).

In intervention clusters, community health workers (accredited social health activists, India; agentes polivalentes elementares, Mozambique; nurses, midwives, community health extension workers, and health assistants, Nigeria; and lady health workers, Pakistan) were trained to provide mobile health–guided contacts that were pregnancy hypertension-oriented, antenatal and postpartum care at home (India, Pakistan, and Mozambique) or at a primary health center (Nigeria). Women in control clusters received usual care, advocated by the World Health Organization. In none of the study countries did community health workers usually either manage pregnancy hypertension or carry with them blood pressure measurement devices or antihypertensive medication.

Digital health-guided clinical assessments were recommended every 4 weeks until 28 weeks of gestation, every 2 weeks from 28 to 35 weeks, weekly from 36 weeks until delivery, once within 24 hours of birth, and postnatally around postpartum days 3, 7, and 14; in Nigeria, visits were opportunistic when women attended the primary health center [16,17].

For generalizability, as sBP can be identified by the return of a radial pulse without the requirement for a stethoscope, the CLIP trial protocol selected a sBP of at least 140 mm Hg as the threshold at which community health workers would respond by initiating referral. In addition, community health workers were directed by the app to either administer oral methyldopa 750 mg for sBP of 160 mm Hg or higher; administer intramuscular magnesium sulfate 10 g for suspected severe preeclampsia (miniPIERS [mini Pre-eclampsia Integrated Estimate of Risk] for an adverse maternal outcome of at least 25%, severe systolic hypertension [at least 160 mm Hg], eclampsia, stroke, or vaginal bleeding); or refer the woman to a comprehensive emergency obstetric care facility for suspected preeclampsia or increased risk of stillbirth (4+ dipstick proteinuria value, or absent fetal movements for at least 12 h) [10,14].

### The PRECISE Observational Cohorts

Briefly, in each participating country, 2 cohorts of women were recruited to optimize the identification of social, clinical, and biomarker determinants of the placental disorders of pregnancy, with purposeful sampling of both urban-dwelling and rural-dwelling women [18].

First, unselected pregnant women planning to give birth in the facility (primary health center) were approached and recruited at the time of their booking visit for antenatal care [18]. Consenting women answered an in-depth questionnaire regarding social determinants and clinical history, underwent a targeted examination (eg, biometry, BP, and pulse oximetry), and provided biological samples (blood, urine, and vaginal swabs). Participants were followed up (1) for a second antenatal research visit in the third trimester, at least 4 weeks after the first visit (questionnaire, targeted examination, and phlebotomy); (2) the birth episode (both antepartum and postpartum; questionnaire, targeted examination, phlebotomy, vaginal swabs, placental, membrane, and cord blood samples); and (3) 6 weeks to 6 months postpartum (with their infant, if alive; questionnaire, targeted maternal and infant examination, phlebotomy, vaginal swabs, and heel-prick samples).

Second, for comparison and to understand the lives and biology of sub-Saharan African nonpregnant women of reproductive age (16‐49 y); such women were identified as either accompanying the pregnant women described in Table 1 or attending for family planning services (Kenya and Mozambique) or recruited in their homes by research staff accompanying the district health surveillance system team (the Gambia). Consenting women answered an in-depth questionnaire regarding social determinants and clinical history, underwent a targeted examination (eg, biometry, BP, and pulse oximetry), and provided biological samples (blood, urine, and vaginal swabs) [18].

**Table 1.** Characteristics and pregnancy outcomes (where relevant) of the study cohorts.

Characteristics	CLIP^a^ trials (n=32,053)	PRECISE^b^ pregnancy cohorts (n=6932)	PRECISE WRA^c^ cohorts (n=1825)
Age (y)^d^, median (IQR)	25 (22-30)	25 (21-30)	28 (23-34)
Nulliparous, n (%)	8782 (27.4)	2112 (30.47)	274 (15.01)
Marital status, n (%)			
Never married or single	1029 (3.21)	1148 (16.56)	445 (24.38)
Married or cohabiting	26 032 (981.22)	5684 (82)	1295 (70.96)
Separated or divorced	208 (0.65)	68 (0.98)	58 (3.18)
Widowed	128 (0.4)	32 (0.46)	27 (1.48)
No formal education, n (%)	14,926 (46.57)	1243 (17.93)	500 (27.4)
Probability of poverty, median (IQR)	—^e^	20.3 (6.1-36.9)	31.7 (13.9-51.3)
GA^f^ at enrollment (wk), median (IQR)	22 (15-29)	21 (15-27)	—
Women with birth outcome data			
Outcome (n=23,265 for CLIP; n=5832 for PRECISE)			
GA at delivery (wk), median (IQR)	39.0 (37.0-40.4)	39.0 (37.3-40.4)	—
Maternal death, n (%)	43 (0.18)	8 (0.14)	—
Miscarriage or TOP^g^ (<20 wk), n (%)	601 (2.58)	21 (0.36)	—
Stillbirth (≥20 wk), n (%)	819 (3.52)	182 (3.12)	—
Neonatal death, n (%)	862 (3.71)	27 (0.46)	—

a CLIP: Community-Level Interventions for Preeclampsia.

b PRECISE: Pregnancy Care Integrating Translational Science, Everywhere.

c WRA: nonpregnant women of reproductive age.

d Maternal age standardized to age at expected date of delivery for pregnant women.

e Not applicable.

f GA: gestational age.

g TOP: termination of pregnancy.

Neither patients nor the public were involved in the design, or conduct, or reporting, or dissemination plans of this audit.

### BP Measurement Protocol

The Microlife BP 3AS1-2 device was used in the Indian, Mozambican, and Pakistani CLIP trials, and its successor, the CRADLE VSA, in the Nigerian CLIP pilot trial, and the PRECISE pregnancy and nonpregnancy cohorts. Both devices are inflated manually and use the steady deflationary phase to detect BP, algorithmically calculating systolic and diastolic values from the measured mean arterial pressure and displaying the sBP and dBP and heart rate values on digital read-outs on the front of the device.

The research staff (community health workers in CLIP and nurses in PRECISE) were trained to have women rest seated for 5 minutes, and then to measure BP in a standardized fashion, with the left arm supported and the cuff at the level of the heart, at least twice. All BP readings were entered manually into a study-specific digital platform, which averaged them as the BP for that visit; the first and second readings were averaged unless they were more than 10 mm Hg different, in which instance a third reading was requested by the study digital platform, and the second and third readings were averaged [12].

### Statistical Analyses

All BP readings were used for the analyses.

We determined the incremental change in sBP and dBP between the first and second measurements, the second and third measurements, and the first and third measurements, if a third measurement was taken.

Individual BP value frequencies were displayed for both sBP and dBP for each study site, cumulatively for both the CLIP trials and the PRECISE cohorts, and cumulatively for the combined dataset. This was to identify if there was any evidence of terminal digit preference and threshold avoidance. Through that step, evidence of terminal digit preference was confirmed, overreporting of the dBP value of 62 mm Hg became apparent (see the “Results” section), and, in the CLIP dataset, suspicion of threshold avoidance at 140 mm Hg systolic was observed.

We compared the directly observed counts for dBP of 62 mm Hg versus the averaged count for dBP of 62 mm Hg from the duplicate measurement (ie, 1 and 2 of two measurements or 2 and 3 of three measurements) using a Fisher exact test and the Koopman asymptotic score to calculate 95% CI for the relative risk. The percentage of values ending in “0” and “5” was calculated and compared with the expected value of 10% (assuming a uniform distribution of digits) using both raw and averaged measurements using a 1-sample test for proportion.

To assess threshold avoidance in the CLIP dataset (where an intervention was associated with systolic hypertension), we fit a segmented regression model to the number of BP measurements per unit. Specifically, we used a Poisson model with the following covariates: BP level, threshold (above and below 140 mm Hg), and an interaction between these terms. We display fitted values from these models graphically and use a likelihood ratio test comparing models with and without the segmentation to assess evidence of the possible discontinuity corresponding to threshold avoidance. We compared these results with the CLIP dBP values and PRECISE sBP and dBP values, where no relevant clinical decision thresholds were set.

All analyses were performed in R statistical software version 4.0.3 (R Foundation for Statistical Computing). *P*<.01 was used for statistical significance to adjust for multiple comparisons.

### Ethical Considerations

The CLIP trials were approved by the University of British Columbia Research Ethics Board (H12-03497) and within each country (MDC/IECHSR/2013‐14/A, KLE University, Belgavi, India; 2590-Obs-ERC-13, Aga Khan University, Karachi, Pakistan; 219/CNBS/13, the Mozambique Ministry of Health, National Bioethics Committee for Health, Maputo, Mozambique; and OOUTH/DA.326/T/1/, Olabisi Onabanjo University Teaching Hospital, Sagamu, Nigeria). The PRECISE study was approved by King’s College London (HR-17/18‐7855) and within each country (2018/REC-74, Aga Khan University Hospital, Nairobi, Kenya; SCC 1619, the Gambia Government/the Medical Research Council, the Gambia Joint Committee, Banjul, the Gambia; and 545/CNBS/18, the Mozambique Ministry of Health, National Bioethics Committee for Health, Maputo, Mozambique). All women provided signed informed consent before taking part in either the CLIP trials or the PRECISE study. All data were deidentified and anonymized. CLIP trial participants were not compensated, other than support for urgent transfers to health facilities when complications were noted or per protocol when risks were identified. PRECISE participants were supported to attend study visits (transport and meals) and were supported for urgent transfers to health facilities as indicated by clinical concerns.

## Results

Data were available for 51,875 eligible women, who were recruited from communities and primary health centers in 6 countries (the Gambia, India, Kenya, Mozambique, Nigeria, and Pakistan), with a median of 2841 (IQR 610‐7862) women per included cohort. In total, 911,500 discrete sBP or dBP values were available for analysis.

Some individual-level characteristics of the participating women varied between cohorts (Table 1; Table S2 in Multimedia Appendix 1). Women tended to be in their mid-to-late 20s, were variably nulliparous (range 6.4%-39.5%), and, if pregnant, were recruited from 10.8 to 42.6 weeks’ gestation. Pregnant participants generally delivered at term. Miscarriage and termination of pregnancy rates reflect generally late booking for antenatal care, as most miscarriages occur before 12 weeks of gestation. Maternal mortality varied from 0% to 0.27%, stillbirths from 2.2% to 5.1%, and neonatal deaths from 0.32% to 5% (Table 1; Table S2 in Multimedia Appendix 1).

**Figure 1.**
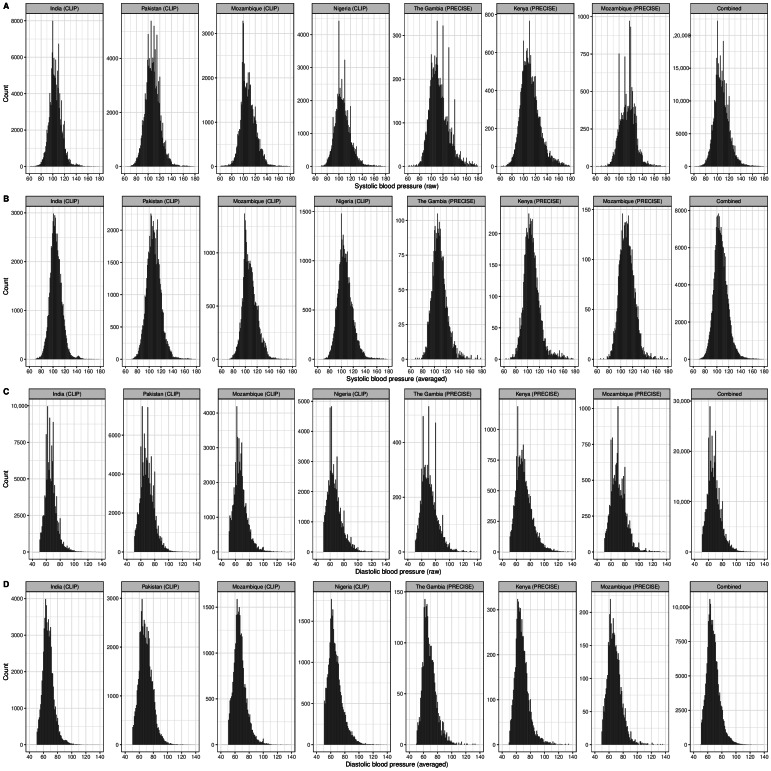
Blood pressure value profiles from the CLIP trials and PRECISE Network. (A) Raw systolic blood pressure values; (B) raw diastolic blood pressure values; (C) averaged systolic blood pressure values; (D) averaged diastolic blood pressure values. CLIP: Community-Level Interventions for Preeclampsia; PRECISE: Pregnancy Care Integrating Translational Science, Everywhere.

First BP measurements tended to be higher compared with subsequent measurements, but not consistently (Table 2; Table S3 in Multimedia Appendix 1). 25,693 of 183,504 (14%) measurements were taken in triplicate. The raw and averaged BP profiles from the CLIP trials and PRECISE Network (Figure 1) examined BP distributions, terminal digit preference, and the unanticipated rate of dBP values at 62 mm Hg.

**Table 2.** Characteristics of blood pressure values.

Characteristics	CLIP^a^ trials (n=43,118 pregnancies)	PRECISE^b^ pregnancy cohorts (n=6932 pregnancies)	PRECISE WRA^c^ cohorts (n=1825 women)
BP^d^ values, n/N (%)			
2 values	157,811/183,304 (86)	17,249/19,625 (87.89)	1389/1822 (76.23)
3 values	25,693/183,304 (14)	2376/19,625 (12.11)	433/1822 (23.77)
Differences in BP values (mm Hg), median (IQR)			
sBP1^e^^,f^-sBP2^g^	1 (–2 to 5)	3 (0 to 8)	2 (–2 to 6)
sBP1-sBP3^h^	1 (–2 to 5)	3 (0 to 8)	2 (–2 to 6)
sBP2-sBP3	0 (–4 to 4)	0 (–4 to 5)	–1 (–9 to 6)
dBP1^f^^,^^i^-dBP2	1 (–2 to 4)	2 (0 to 6)	1 (–1 to 4)
dBP1-dBP3	1 (–2 to 4)	2 (0 to 6)	1 (–1 to 4)
dBP2-dBP3	0 (–3 to 3)	0 (–2 to 4)	0 (–3 to 4)
Terminal digit preference—raw values (sBP), n/N (%)			
Values ending in “0” (N)	57,343/392,759 (14.6)	6393/41,055 (15.57)	468/4513 (10.37)
Values ending in “5” (N)	44,614/392,759 (11.36)	3782/41,055 (9.21)	388/4513 (8.6)
Terminal digit preference—raw values (dBP), n/N (%)			
Values ending in “0”	58,677/392,319 (14.96)	6245/41,048 (15.21)	409/4079 (10.03)
Values ending in “5”	45,470/392,319 (11.59)	3699/41,048 (9.01)	406/4079 (9.95)
Terminal digit preference—averaged values (sBP), n/N (%)			
Values ending in “0”	18,832/182,636 (10.31)	869/10,911 (7.96)	130/1801 (7.22)
Values ending in “5”	17,159/182,636 (9.4)	868/10,911 (7.96)	139/1801 (7.72)
Terminal digit preference—averaged values (dBP), n/N (%)			
Values ending in “0”	18,808/182,637 (10.3)	819/10,911 (7.51)	149/1801 (8.27)
Values ending in “5”	17,302/182,637 (9.47)	847/10,911 (7.76)	142/1801 (7.88)
Algorithm issues—raw values, n/N (%)			
dBP=62 mm Hg	26,474/392,319 (6.75)	2281/41,048 (5.56)	174/4079 (4.27)
Algorithm issues—averaged values, n/N (%)			
dBP=62 mm Hg	8774/182,637 (4.8)	495/10,911 (4.54)	41/1801 (2.28)

a CLIP: Community-Level Interventions for Preeclampsia.

b PRECISE: Pregnancy Care Integrating Translational Science, Everywhere.

c WRA: nonpregnant women of reproductive age.

d BP: blood pressure.

e sBP: systolic blood pressure.

f BP1: first value.

g BP2: second value.

h BP3: third value (if taken).

i dBP: diastolic blood pressure.

There was clear evidence of terminal digit preference for raw values ending in “0” (129,539/911,500, 14.2%) and “5” (98,359/911,500, 10.8%) versus 10% expected (Table 2; Table S3 in Multimedia Appendix 1; *P*<.001 for both “0” and “5”); these were obviated in the averaged values for “0” (39,607/390,697, 10.1%) and “5” (36,457/390,697, 9.3%).

**Figure 2.**
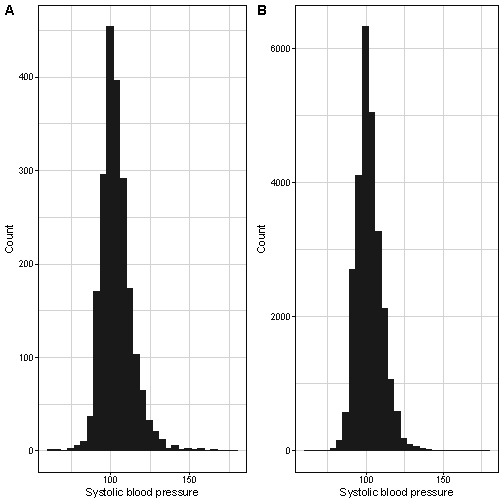
Systolic values corresponding to diastolic blood pressure of 62 mm Hg in (A) CLIP (Community-Level Interventions for Preeclampsia; 55 and 31 of 26,474 ≥140 mm Hg and ≥160 mm Hg, respectively) and (B) PRECISE (Pregnancy Care Integrating Translational Science, Everywhere; 15 and 2 of 2094 ≥140 mm Hg and ≥160 mm Hg, respectively).

There was a greater than expected frequency of dBP values of 62 mm Hg (raw observations: 28,929/437,446, 6.6% vs averaged observations: 9310/195,349, 4.8%, *P*<.01; relative risk 1.094, 95% CI 1.09‐1.10; Table 2; Table S3 in Multimedia Appendix 1; Figure 1). These errors in data recording into digital platforms were largely obviated by averaging the sBP and dBP values (first and second or, if required, second and third; Table 2; Table S3 in Multimedia Appendix 1; Figure 1). The sBP values corresponding to these 62 mm Hg diastolic values varied considerably and were approximately normally distributed (Figure 2); in both cohorts, very few of these values were at least 140 mm Hg (53, 0.2% in CLIP; 17, 0.7% in PRECISE) or at least 160 mm Hg (0.1% in both cohorts).

**Figure 3.**
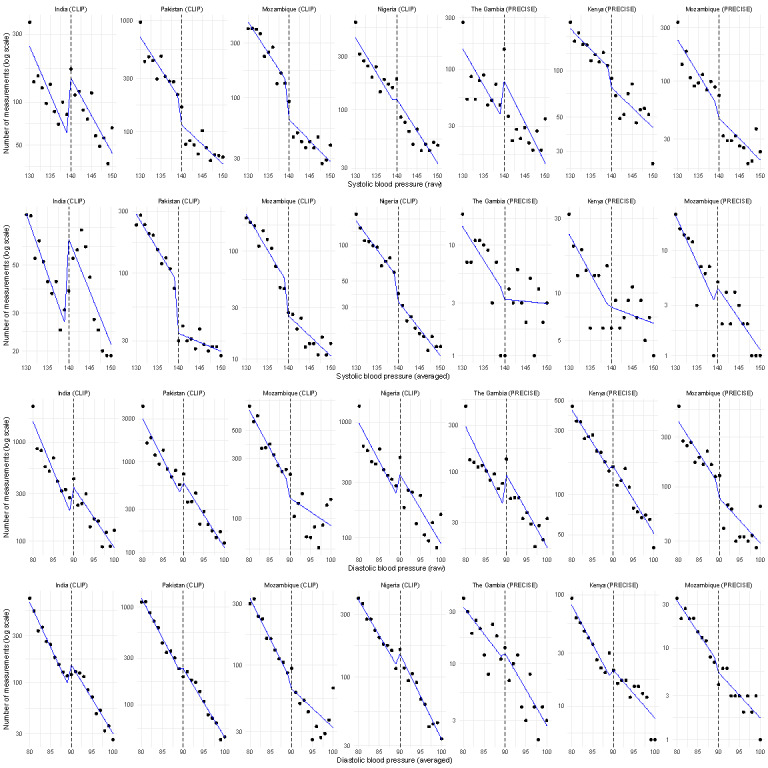
Threshold preference and avoidance in the CLIP (Community-Level Interventions for Preeclampsia) trials and PRECISE (Pregnancy Care Integrating Translational Science, Everywhere) network. The blue lines are fitted values from segmented Poisson regression models.

For sBP readings, there was evidence of both threshold (140 mm Hg) preference (India; *P*=.02) and threshold avoidance in Mozambique (CLIP, *P*=.007; PRECISE, *P*=.006) and Pakistan (*P*<.001), but for neither the Gambia (*P*=.31), Kenya (*P*=.85), nor Nigeria (*P*=.50; Figure 3). These observations were modified by averaging values (the Gambia *P*=.05; India *P*=.47; Kenya *P*=.05; Mozambique CLIP *P*=.01, PRECISE *P*=.35; Nigeria *P*=.76; and Pakistan *P*<.001; Figure 3). For dBP readings, there was evidence of threshold (90 mm Hg) preference (India *P*<.001; Pakistan *P*<.001) and avoidance (Mozambique CLIP *P*<.001, PRECISE *P*=.008), but neither for the Gambia (*P*=.11), Kenya (*P*=.70), nor Nigeria (*P*=.05; Figure 3). Generally, these observations were modified by averaging values (the Gambia *P*=.44; India *P*<.001; Kenya *P*=.06; Mozambique CLIP *P*<.001, PRECISE *P*=.49; Nigeria *P*=.08; Pakistan *P*=.03; Figure 3).

## Discussion

### Principal Results

Using BP data from 51,875 prospectively recruited eligible women living in 5 low- and middle-income countries, 911,500 discrete sBP or dBP values were analyzed in this study. Our protocol of repeating (up to 3 times) and averaging BP measurements (values 1 and 2 of 2, or 2 and 3 of 3) clearly improves the quality of data and clinical guidance that are derived from use of these 2 devices. This was true for the terminal digit preference and the algorithmic issue that resulted in the excess of dBP values of 62 mm Hg, but not for threshold avoidance.

### Comparison With Prior Work

Reassuringly for clinical use of these two devices, 62 mm Hg diastolic readings were rarely (≥0.7%) associated with a sBP value of at least 140 mm Hg, and only 0.1% were associated with systolic readings of at least 160 mm Hg that increase the risk for fatal intracranial bleeding [19]. In addition, it might be useful to assess whether or not the 62 mm Hg issue is shared between other Microlife automated and semiautomated BP devices.

Within the CLIP trials, the addition of the traffic light display may underlie why there was no evidence of threshold avoidance in the CLIP Nigeria pilot trial. The CLIP Nigeria pilot trial was the sole part of the CLIP endeavor that used the then-new CRADLE VSA device [9], compared with the BP 3AS1-2 device used in the other CLIP trials. In India (threshold preference), and Mozambique and Pakistan (threshold avoidance) were clearly seen for systolic, but less so diastolic, hypertension, as the protocol was sBP-driven [6-8,10]. The display of an amber or red light that was visible to others in the room when the BP was taken may have provided important shared information to guide shared decision-making about initiating transfers of care and, in some cases, methyldopa and magnesium sulfate therapy. However, both systolic threshold preference and threshold avoidance were observed in Gambian and Mozambican, but not Kenyan, PRECISE cohort. Diastolic preference and avoidance were both seen, but were of lower magnitude than for sBP values. It would be interesting to understand why the Gambian research staff and the Indian Accredited Social Health Activist workers preferred a value diagnostic of hypertension, while Mozambican research staff and *Agentes Polivalentes Elementares* and Pakistani lady health workers displayed threshold avoidance. Were there differences in the training about the importance of detecting hypertension, or were there concerns about the additional workload associated with making such a diagnosis [15,20-22]?

Previously in the CLIP dataset, we have identified that lowering both sBP and dBP thresholds by 5 mm Hg (ie, 135 mm Hg and 85 mm Hg, respectively, for nonsevere hypertension; and 155 mm Hg and 105 mm Hg, respectively, for severe hypertension), for measurements taken in community settings would improve the detection of pregnant women at risk of the complications associated with pregnancy hypertension, especially preeclampsia [23].

### Strengths and Limitations

The strengths of this study include prospective data collection by trained individuals, a shared standardized BP measurement protocol, geographical collection of data from women living in 2 South Asian and 4 sub-Saharan countries (2 from west, and one each from east and southern Africa), and its large sample size. The two study designs, cluster randomized controlled trials (CLIP) and prospective observational cohort (PRECISE), provided additional insights into the role of threshold avoidance with and without the traffic light display introduced in the CRADLE VSA device.

Our limitations include the absence of a wirelessly connected device for comparison, by which means we could have tested the hypothesis that the terminal digit preference and threshold avoidance could be overcome by wireless data collection onto digital health platforms. In addition, we were unable to examine the reasons behind the differences in response to BP thresholds (ie, preference: the Gambia, India vs avoidance: Mozambique, Pakistan vs none: Kenya, Nigeria) between study sites. Such insights would help to overcome threshold avoidance, in particular, in the interim before wireless connectivity between BP devices and digital health platforms becomes routine. Threshold avoidance is a safety signal as women at risk may not have been referred when appropriate.

### Conclusions

In summary, the BP 3AS1-2 and CRADLE VSA have been used reliably in a number of other clinical trials and studies with largely positive results [24-27]. They have provided generally reliable, low-cost BP monitors that can be safely deployed from households to facilities in low- and middle-income countries. The addition of the traffic lights in the CRADLE VSA appears to have reduced the risk of threshold avoidance. However, in this study, we have identified opportunities for further improvements that relate to terminal digit preference and the algorithm issues around 62 mm Hg. In the interim, clinicians and fellow researchers should use our protocol for using these devices 2 to 3 times to reduce errors.

## Supplementary material

10.2196/73307 Multimedia Appendix 1 Research group members and detailed cohort and blood pressure descriptions.

10.2196/73307 Checklist 1 STROBE checklist.

## Abbreviations

BP: blood pressure
CLIP: Community-Level Interventions for Preeclampsia
dBP: diastolic blood pressure
PRECISE: Pregnancy Care Integrating Translational Science, Everywhere
sBP: systolic blood pressure
STROBE: Strengthening the Reporting of Observational Studies in Epidemiology
VSA: Vital Signs Alert
