# Supplementary material for: Terminal Digit Preference and Threshold Avoidance in Digital Blood Pressure Measurements During Pregnancy: Secondary Analysis of Data From the CLIP and PRECISE Cohorts
Source: JMIR Mhealth Uhealth. 2026 Jun 3;14:e73307. doi: 10.2196/73307 (PMC13232918; doi:10.2196/73307)
Supplement: Multimedia Appendix 1 [file mhealth-v14-e73307-s001.pdf]

Supplementary Table 1

CLIP Trials and PRECISE Study Groups

**CLIP Trials Study Group**
CLIP Mozambique<br>Esperança Sevene, Eusébio Macete, Khátia Munguambe, Charfudin Sacoor, Anifa Vala, Helena Boene, Felizarda Amose, Rosa Pires, Zefanias Nhamirre, Marta Macamo, Rogério Chiaú, Analisa Matavele, Faustino Vilanculo, Ariel Nhancolo, Silvestre Cutana, Ernesto Mandlate, Salésio Macuacua, Cassimo Bique, Sibone Mocumbi, Emília Gonçálves, Sónia Maculuve, Ana Ilda Biz, Dulce Mulungo, Orvalho Augusto, Paulo Filimone, Vivalde Nobela, Corsino Tchavana, Cláudio Nkumbula
CLIP Pakistan<br>Rahat Qureshi, Zulfiqar A Bhutta, Zahra Hoodbhoy, Farrukh Raza, Sana Sheikh, Javed Memon, Imran Ahmed, Amjad Hussain
CLIP India<br>Mrutunjaya B Bellad, Umesh S Charantimath, Shivaprasad S Goudar, Geetanjali M Katageri, Avinash J Kavi, Amit P Revankar, Ashalata A Mallapur, Umesh Y Ramdurg, Shashidhar G Bannale, Vaibhav B Dhamanekar, Geetanjali I Mungarwadi, Narayan V Honnungar, Bhalachandra S Kodkany, Anjali M Joshi, Uday S Kudachi, Sphoorthi S Mastiholi, Chandrappa C Karadiguddi, Gudadayya S Kengapur, Namdev A Kamble, Keval S Chougala
University of British Columbia and King's College London<br>Jeffrey N Bone, Dustin T Dunsmuir, Sharla K Drebit, Chirag Kariya, Mai-Lei Woo Kinshella, Tang Lee, Jing Li, Mansun Lui, Beth A Payne, Diane Sawchuck, Sumedha Sharma, Andrew H Shennan, Domena K Tu, Marianne Vidler, Ugochi V·Ukah, Laura A Magee, Peter von Dadelszen
**CLIP Trial Steering Committee**
J Mark Ansermino, Ana Pilar Betrán, Richard Derman, Shafik Dharamsi, France Donnay, Sharla Drebit, Guy Dumont, Susheela M Engelbrecht, Veronique Fillipi, Tabassum Firoz, William Grobman, Marian Knight, Ana Langer, Simon Lewin, Gwyneth Lewis, Craig Mitton, Nadine Schuurman, Andrew H Shennan, Joel Singer, James G Thornton, Hubert Wong
**CLIP Trial Executive Committee**
Olalekan O Adetoro, Mrutunjaya M Bellad, Zulfiqar A Bhutta, Peter von Dadelszen, Shivaprasad S Goudar, Jerker Liljestrand, Laura A Magee, Ashalata A Mallapur, Khátia Munguambe, Beth A Payne, Rahat Qureshi, Charfudin Sacoor, Esperança Sevene, Sumedha Sharma, John O Sotunsa, Marianne Vidler
**CLIP Data Safety and Monitoring Board (DSMB)**
Romano Nkumbwa Byaruhanga, Brian Darlow, Eileen Hutton, Mario Merialdi, Lehana Thabane
**PRECISE Study Group**
PRECISE The Gambia<br>Umberto D'Alessandro, Anna Roca, Hawanatu Jah, Abdoulie Bah, Yorro Bah, Brahima Diallo, Gibril Gabbidon, Lawrence Gibba, Yahaya Idris, Fatoumata Kongira, Melisa Martinez-Alvarez, Modou FS Ndure, Baboucarr Njie, Andrew Prentice, Abdul Sesay, Sambou Suso, Fatima Touray

PRECISE Kenya<br>Marleen Temmerman, Angela Koech, Consolata Juma, Thomas Mendy, Moses Mukhanya, Isaac Mwaniki, Grace Mwashigadi, Joseph Mutunga, Emily Mwadime, Marvin Ochieng, Patricia Okiro, Geoffrey Omuse, Onesmus Wanje
PRECISE Mozambique<br>Esperança Sevene, Anifa Vala, Helena Boene, Carla Carrilho, Salesio Macuacua, Sonia Maculuve, Inacio Mandomando, Lazaro Quimice, Corssino Tchavana
Midlands State University<br>Prestige Tatenda Makanga, Liberty Makacha, Reason Mlambo
King's College London, University of Oxford, London School of Hygiene & Tropical Medicine, St George's, University of London, Imperial College London, University of British Columbia<br>Peter von Dadelszen, Laura A. Magee, Rachel Craik, Marie-Laure Volvert, Hiten Mistry, Meriel Flint-O'Kane, Amber Strang, Marina Daniele, Lucilla Poston, Rachel Tribe, Sophie Moore, Tatiana Salisbury, Aris Papageorghiou, Alison Noble, Rachel Craik, Hannah Blencowe, Veronique Filippi, Joy Lawn, Matt Silver, Joseph Akuze, Ursula Gazeley, Ben Barratt, Judith Cartwright, Guy Whitley, Sanjeev Krishna, Marianne Vidler, Jing (Larry) Li, Jeffrey N Bone, Mai-Lei (Maggie) Woo Kinshella, Domena Tu, Ash Sandhu, Kelly Pickerill
Donna Russell Consulting<br>Donna Russell

Table 2

Supplementary Table 2 Characteristics and pregnancy outcomes (where relevant) of the study cohorts

	CLIP trials					PRECISE pregnancy cohorts				PRECISE WRA cohorts			
	**India (N=7055)**	**Mozambique (N=4809)**	**Nigeria (N=8790)**	**Pakistan (N=11,399)**	**Combined (N=32,055)**	**The Gambia (N=1251)**	**Kenya (N=3584)**	**Mozambique (N=2097)**	**Combined (N=6932)**	**The Gambia (N=610)**	**Kenya (N=609)**	**Mozambique (N=606)**	**Combined (N=1825)**
Age (years)*	23 [20, 25]	23 [19, 30]	27 [23, 31]	28 [25, 30]	25 [22, 30]	26 [22, 31]	26 [23, 31]	23 [19, 29]	25 [21, 30]	28 [22, 36]	27 [23, 33]	28 [23, 35]	28 [23, 34]
Nulliparous (N)	2586 (36.7%)	1478 (30.9%)	2107 (66.4%)	2611 (23.0%)	8782 (33.3%)	237 (18.9%)a	1047 (29.2%)	828 (39.5%)	2112 (30.5%)	175 (28.7%)	39 (6.4%)	60 (9.9%)	274 (15.0%)
Marital status													
Never married/ single	0 (0.0%)	952 (20.6%)	77 (1.77%)	0 (0.00%)	1029 (3.76%)	22 (1.8%)	220 (6.1%)	906 (43.2%)	1148 (16.6%)	97 (15.9%)	55 (9.0%)	293 (48.3%)	445 (24.4%)
Married/ Co-habiting	7055 (100%)	3345 (72.4%)	4264 (98.2%)	11,368 (99.9%)	26 032 (95.0%)	1223 (97.8%)	3282 (91.6%)	1179 (56.2%)	5684 (82.0%)	496 (81.3%)	503 (82.6%)	296 (48.8%)	1295 (71.0%)
Separated/ Divorced	0 (0.0%)	207 (4.5%)	0 (0.0%)	1 (0.01%)	208 (0.76%)	6 (0.5%)	55 (1.5%)	7 (0.3%)	68 (1.0%)	12 (2.0%)	42 (6.9%)	4 (0·7%)	58 (3·2%)
Widowed	0 (0.0%)	119 (2.6%)	2 (0.05%)	7 (0.06%)	128 (0.47%)	0 (0.0%)	27 (0.8%)	5 (0.2%)	32 (0.5%)	5 (0.8%)	9 (1.5%)	13 (2.1%)	27 (1.5%)
No formal education	4123 (58.4%)	2807 (58.4%)	3112 (71.7%)	2610 (23·0%)	14 926 (54.1%)	797 (63.7%)	337 (9.4%)	109 (5.2%)	1243 (17.9%)	373 (61.1%)	62 (10.2%)	65 (10.7%)	500 (27.4%)
Probability of poverty (%)	-	-	-	-	-	46.9 [27.9, 58.6]	13.9 [4.6, 30.0]	21.4 [7.2, 31.7]	20.3 [6.1, 36.9]	46.9 [34.8, 56.0]	17.8 [6.1, 36.9]	28.8 [8.5, 31.7]	31.7 [13.9, 51.3]
GA at enrolment (weeks)	12 [9, 18]	27 [22, 32]	27 [21, 33]	22 [16, 28]	22 [15, 29]	19 [15, 23]	21 [16, 25]	21 [16, 26]	21 [15, 27]	-	-	-	-
**Women with birth outcome data**													
	**India (N=7055)**	**Mozambique (N=4809)**	**Nigeria (N=8790)**	**Pakistan (N=11399)**	**Combined (N=32,265)**	**The Gambia (N=1208)**	**Kenya (N=2744)**	**Mozambique (N=1880)**	**Combined (N=5832)**	**The Gambia**	**Kenya**	**Mozambique**	**Combined**
GA at delivery (weeks)	39.0 [38.0, 40.0]	39.3 [37.3, 41.0]	-	38.6 [36.1, 40.7]	39.0 [37.0, 40.4]	39.0 [37.0, 40.4]	39.0 [37.1, 40.4]	39.1 [37.9, 40.6]	39.0 [37.3, 40.4]	-	-	-	-
Maternal death (N)	7 (0.11%)	7 (0.17%)	-	29 (0.28%)	43 (0.21%)	3 (0.25%)	0 (0.0%)	5 (0.18%)	8 (0.14%)	-	-	-	-
Miscarriage/ TOP <20 weeks (N)	376 (6.1%)	49 (1.2%)	-	176 (1.7%)	601 (2.9%)	12 (0.99%)	4 (0.15%)	5 (0.27%)	21 (0.36%)	-	-	-	-
Stillbirth ≥20 weeks (N)	171 (3.1%)	138 (3.3%)	-	510 (4.9%)	819 (4.1%)	62 (5.1%)	59 (2.2%)	61 (3.2%)	182 (3.1%)	-	-	-	-
Neonatal death (N)	161 (3.0%)	138 (3.4%)	-	563 (5.6%)	862 (4.4%)	5 (0.40%)	16 (0.58%)	6 (0.32%)	27 (0.46)	-	-	-	-

Data are n (%) or median (IQR)· NA=not applicable· *maternal age standardised to age at expected date of delivery for pregnant women· CLIP=Community-Level Interventions for Pre-eclampsia; GA=gestational age; PRECISE=PREgnancy Care Integrating translational Science, Everywhere; TOP=termination of pregnancy; WRA=non-pregnant women of reproductive age

Supplementary Table 3 Characteristics of blood pressure values

	CLIP trials					PRECISE pregnancy cohorts				PRECISE WRA cohorts			
	India (N=7839 pregnancies)	Mozambique (N=7930 pregnancies)	Nigeria (N=7114 pregnancies)	Pakistan (N=20,235 pregnancies)	Combined (N=43,118 pregnancies)	The Gambia (N=1251 pregnancies)	Kenya (N=3584 pregnancies)	Mozambique (N=2097 pregnancies)	Combined (N=6932 pregnancies)	The Gambia (N=610 pregnancies)	Kenya (N=609 women)	Mozambique (N=606 women)	Combined (N=1825 women)
BP values (N)													
2 values	57 627	23 929	26 809	49 344	157 811	2950	8014	6285	17 249	473	478	438	1389
3 values	6361	4723	5890	8821	25 693	463	1098	815	2376	136	129	168	433
Differences in BP values (mmHg)													
sBP1 - sBP2	2 [-2, 4]	2 [-1, 5]	1 [-1, 5]	1 [-3, 5]	1 [-2, 5]	2 [0, 6]	3 [0, 7]	4 [0, 9]	3 [0, 8]	2 [-2, 6]	2 [-1, 6]	2 [-1, 6]	2 [-2, 6]
sBP1 - sBP3	2 [-2· 4]	2 [-1, 5]	1 [-1, 5]	1 [-3, 5]	1 [-2, 5]	2 [0, 6]	3 [0, 7]	4 [0, 9]	3 [0, 8]	2 [-2, 6]	2 [-1, 6]	2 [-1, 6]	2 [-2, 6]
sBP2 - sBP3	0 [-4, 3]	0 [-3, 4]	0 [-3, 4]	-1 [-6, 4]	0 [-4, 4]	1 [-3, 8]	-1 [-5, 3]	1 [-2, 5]	0 [-4, 5]	1 [-5, 6]	-1 [-6, 3]	-1 [-10, 6]	-1 [-9, 6]
dBP1 - dBP2	1 [-2, 4]	2 [-1, 4]	1 [-1, 4]	0 [-3, 4]	1 [-2, 4]	1 [0, 4]	2 [0, 5]	3 [0, 9]	2 [0, 6]	1 [-2, 4]	1 [-1, 4]	1 [-2, 4]	1 [-1, 4]
dBP1 - dBP3	1 [-2, 4]	2 [-1, 4]	1 [-1, 4]	0 [-3, 4]	1 [-2, 4]	1 [0, 4]	2 [0, 5]	3 [0, 9]	2 [0, 6]	1 [-2, 4]	1 [-1, 4]	1 [-2, 4]	1 [-1, 4]
dBP2 - dBP3	0 [-4, 4]	0 [-3, 4]	0 [-2, 3]	0 [-5, 3]	0 [-3, 3]	0 [-2, 4]	0 [-3, 3]	1 [-2, 5]	0 [-2, 4]	0 [-3, 3]	1 [-4, 4]	0 [-4, 4]	0 [-3, 4]
Terminal digit preference – raw values (sBP)													
Values ending in '0' (N)	20,220/134,258 (15.1%)	8174/62,108 (13.2%)	12,220/71,241 (17.2%)	16,729/125,152 (13.4%)	57,343/392,759 (14.6%)	1319/7069 (18.7%)	2252/18,970 (11.9%)	2822/15,016 (18.8%)	6393/41,055 (15.6%)	133/1357 (9.8%)	161/1343 (12.0%)	174/1813 (9·6%)	468/4513 (10.4%)
Values ending in '5' (N)	15,441/134,258 (11.5%)	5548/62,108 (8.9%)	7641/71,241 (10.7%)	15,984/125,152 (12.8%)	44,614/392,759 (11.4%)	666/7069 (9.4%)	1896/18,970 (10.0%)	1220/15,016 (8.1%)	3782/41,055 (9.2%)	145/1357 (10.7%)	121/1343 (9.0%)	122/1813 (6.7%)	388/4513 (9.3%)
Terminal digit preference – raw values (dBP)													
Values ending in '0' (N)	21,010/134,221 (15.7%)	6957/61,697 (11.3%)	11,712/71,251 (16.4%)	18,998/125,150 (15.2%)	58,677/392,319 (15.0%)	1368/7063 (19.4%)	2246/18,965 (11.8%)	2631/15,020 (17.5%)	6245/41,048 (15.2%)	120/1354 (8.9%)	139/1343 (10.3%)	150/1382 (10.9%)	409/4079 (10.0%)
Values ending in '5' (N)	16,609/134,221 (12.4%)	6540/61,697 (10.6%)	7255/71,251 (10.2%)	15,066/125,150 (12.0%)	45,470/392,319 (11.6%)	613/7063 (8.7%)	1819/18,965 (9.6%)	1267/15,020 (8.4%)	3699/41,048 (9.0%)	132/1354 (9.7%)	131/1343 (9.8%)	143/1382 (10.3%)	406/4079 (10.0%)
Terminal digit preference – averaged values (sBP)													
Values ending in '0' (N)	6441/63,606 (10.1%)	3209/28,435 (11.3%)	3944/32,484 (12.1%)	6057/63,606 (9.5%)	18,832/182,636 (10.3%)	199/1901 (10.5%)	505/5443 (9.3%)	165/3567 (4.6%)	869/10,911 (8.0%)	52/609 (8.5%)	39/588 (6.6%)	39/604 (6.5%)	130/1801 (7.2%)
Values ending in '5' (N)	6057/63,606 (9.5%)	2666/28,435 (9.4%)	3061/32,484 (9.4%)	5375/58,111 (9.2%)	17,159/182,636 (9.4%)	165/1901 (8.7%)	510/5443 (9.4%)	193/3567 (5.4%)	868/10,911 (8.0%)	64/609 (10.5%)	49/588 (8.3%)	26/604 (4.3%)	139/1801 (7.7%)
Terminal digit preference – averaged values (dBP)													
Values ending in '0' (N)	6897/63,606 (10.8%)	3023/28,435 (10.6%)	3636/32,485 (11.2%)	5252/58,111 (9.0%)	18,808/182,637 (10.3%)	143/1902 (7.5%)	515/5441 (9.5%)	161/3568 (4.5%)	819/10,911 (7.5%)	67/609 (11.0%)	48/588 (8.2%)	34/604 (5.6%)	149/1801 (8.3%)
Values ending in '5' (N)	5964/63,606 (9.4%)	2874/28,435 (10.1%)	3034/32,485 (9.3%)	5430/58,111 (9.3%)	17,302/182,637 (9.5%)	161/1902 (8.5%)	502/5441 (9.2%)	184/3568 (5.2%)	847/10,911 (7.8%)	61/609 (10.0%)	55/588 (9.4%)	26/604 (4.3%)	142/1801 (7.9%)
Algorithm issues – raw values													
dBP=62mmHg	9966/134,221 (7.4%)	4194/61,697 (6.8%)	4833/71,251 (6.8%)	7481/125,150 (6.0%)	26,474/392,319 (6.8%)	424/7063 (6.0%)	1106/18,965 (5.8%)	751/15,020 (5.0%)	2281/41,048 (5.6%(	61/1354 (4.5%)	67/1343 (5.0%)	46/1382 (3.3%)	174/4079 (4.3%)
Algorithm issues – averaged values													
dBP=62mmHg	3474/63,606 (5.5%)	1416/28,435 (5.0%)	1566/32,485 (4.8%)	2318/58,111 (4.0%)	8774/182,637 (4.8%)	106/1902 (5.6%)	277/5441 (5.1%)	112/3568 (3.1%)	495/10,911 (4.5%)	18/609 (3.0%)	14/588 (2.4%)	9/604 (1.5%)	41/1801 (2.3%)

Data are n (%) or median (IQR)· NA=not applicable·

BP=blood pressure; BP1=first value; BP2=second value; BP3=third value (if taken); CLIP=Community-Level Interventions for Pre-eclampsia; dBP=diastolic blood pressure; PRECISE=PREgnancy Care Integrating translational Science, Everywhere; sBP=systolic blood pressure; WRA=non-pregnant women of reproductive age
