# Supplementary material for: Terminal Digit Preference and Threshold Avoidance in Digital Blood Pressure Measurements During Pregnancy: Secondary Analysis of Data From the CLIP and PRECISE Cohorts
Source: JMIR Mhealth Uhealth. 2026 Jun 3;14:e73307. doi: 10.2196/73307 (PMC13232918; doi:10.2196/73307)
Supplement: Checklist 1 [file mhealth-v14-e73307-s002.pdf]

STROBE Checklist

| | Item No. | Recommendation | Page No. | Relevant text from manuscript |
|---|---|---|---|---|
| **Title and abstract** | 1 | (*a*) Indicate the study's design with a commonly used term in the title or the abstract | 1 | The importance of repeated blood pressure measurements using digital devices at single antenatal contacts in pregnancy: data from the Community-Level Interventions for Pre-eclampsia (CLIP) trials and the PREgnancy Care Integrating translational Science, Everywhere (PRECISE) cohorts |
| | | (*b*) Provide in the abstract an informative and balanced summary of what was done and what was found | 3 | **Methods**<br>Two semi-automated, low-cost Microlife blood pressure devices, the BP 3AS1-2 and CRADLE VSA, were used by trained research staff in the Community-Level Interventions for Pre-eclampsia (CLIP) trials and the PREgnancy Care Integrating translational Science, Everywhere (PRECISE) cohorts of pregnant women and non-pregnant women of reproductive age. Both devices are inflated manually and use the steady deflationary phase to detect blood pressure, algorithmically calculating systolic and diastolic values and displaying those values and heart rate on digital read-outs on the device. Trained research staff had women rest, seated for 5 minutes, and measured blood pressure in a standardised fashion (left arm supported and the cuff at heart level), at least twice. All blood pressure readings were entered manually into a digital platform, which averaged them as the blood pressure for that visit; the first and second readings were averaged unless they were more than 10 mmHg different, which triggered a third reading and the second and third readings were averaged. Raw and averaged blood pressure value data were assessed for terminal digit preference and threshold avoidance.<br>**Findings**<br>51,875 participants had their blood pressure measured 438,404 times to derive 204,951 averaged paired systolic blood pressure and diastolic blood pressure values. Us ing raw blood pressure values, there was clear evidence of |

| | | | | |
|---|---|---|---|---|
| | | | | terminal digit preference (of 911,500 values, 129,539 (14·2% vs 10·0%; p<0·001) values ended in a ‘0’, and, 98,359 (10·8% vs 10·0%; p < 0·001) ended in a ‘5’). 28,929 (6·6%) of 437,446 diastolic blood pressure values were 62 mmHg, compared with 9310 (4·8%) of 195,349 from the averaged values (p < 0·001). These errors were obviated by averaging both systolic blood pressure and diastolic blood pressure values. There was evidence of both threshold preference and threshold avoidance in systolic blood pressure (140 mmHg) and diastolic blood pressure (90 mmHg) values in the CLIP trials and the PRECISE observational cohort.<br><br>**Interpretation**<br><br>Given the excess of 62 mmHg values, there is a shared inherent algorithmic error in the calculation of diastolic blood pressure in the BP 3AS1-2 and CRADLE VSA devices. Averaged blood pressure measurements are important to reduce the impact of user errors in manually recording blood pressure values. We recommend that automated and semi-automated blood pressure devices include a Bluetooth function to automatically transfer readings to digital health records to further optimise care. |
| **Introduction** | | | | |
| Background/rationale | 2 | Explain the scientific background and rationale for the investigation being reported | 6 | Pregnancy hypertension (defined as a systolic blood of at least 140 mmHg or a systolic blood pressure of at least 90 mmHg) is associated with approximately 46,000 maternal and 500,000 perinatal deaths annually; more than 99% of those deaths occur in low-income and middle-income countries.[1,2] Pregnancy hypertension is classified as chronic (pre-existing) hypertension, gestational hypertension, and pre-eclampsia.[2] Screening for, detecting, and managing pregnancy hypertension is a core function of antenatal care.[2,3]<br><br>To improve access to blood pressure, and other vital signs measurement, in low-income and middle-income countries, the semi-automated, low-cost Microlife BP 3AS1-2 and CRADLE VSA devices (Widnau, Switzerland) were |

| | | | | |
|---|---|---|---|---|
| | | | | developed, and have been validated in normotensive and hypertensive pregnant women.[4,5] In the context of the Community-Level Interventions for Pre-eclampsia (CLIP) trials, per protocol use of the Microlife BP 3AS1-2 device within a digital health package deployed by community health workers, conducting antenatal contacts in women's homes, cost-effectively improved maternal and perinatal survival.[6-11]<br><br>There are a number of caveats when measuring blood pressure. First, individuals should rest for at least five minutes, and their left arm should be supported, with the cuff at the level of their heart. Second, at least two measurements of blood pressure should be taken, and if there is discordance between the first and second reading, at least one additional measurement should be taken, with the final two values of both systolic blood pressure and diastolic blood pressure being averaged.[12] Blood pressure readings that are not Bluetoothed to a digital application are susceptible to a number of errors, such as terminal digit preference (ie, disproportionately enumerating numbers that end in '0' or '5') and threshold avoidance (ie, enumerating numbers just below the threshold that mandates a clinical response).[13]<br><br>In this study, we studied all the systolic blood pressure and diastolic blood pressure values of the pregnant women who participated in the CLIP trials (including the Nigerian pilot trial) and the non-pregnant women of reproductive age and pregnant women who participated in the PREgnancy Care Integrating translational Science, Everywhere (PRECISE) Network. |
| Objectives | 3 | State specific objectives, including any prespecified hypotheses | 6 | We aimed to (i) determine whether or not repeated blood pressure measurements reduced the presence of terminal digit preference; and (ii) discern whether or not there was evidence of threshold avoidance in the CLIP trials compared with the purely observational PRECISE cohort. |
| **Methods** | | | | |
| Study design | 4 | Present key elements of study design early in the paper | 7 | This is a secondary analysis of data collected in the four countries and 27 intervention clusters of the CLIP cluster randomised controlled trials |

Table 3

| | | | | |
|---|---|---|---|---|
| | | | | (NCT01911494),[14] in India (N = 6, Karnataka State),[6] Pakistan (N = 10, Sindh Province),[7] Mozambique (N = 6, Maputo and Gaza Provinces),[8] and Nigeria (N = 5, Ogun State),[9] and from all participants in the three-country PRECISE Network,[15] in The Gambia (N = 3 primary health centres, Farafenni District), Kenya (N = 2 primary health centres, Kilifi County), and Mozambique (N = 2 primary health centres, Maputo Province). |
| Setting | 5 | Describe the setting, locations, and relevant dates, including periods of recruitment, exposure, follow-up, and data collection | 7-8 | **The CLIP trials**<br><br>In brief, pregnant women (aged 15–49 years in India, Pakistan, and Nigeria, and 12–49 years in Mozambique) were enrolled in the CLIP trials when they first declared their pregnancy and following informed consent.[6-9] The CLIP intervention consisted of community engagement and community health worker-provided mobile health-guided clinical assessment, initial treatment, and referral to facility.[14] The trials were approved by the University of British Columbia Research Ethics Board (H12-03497) and within each country (MDC/IECHSR/2013-14/A, India; 2590-Obs-ERC-13, Pakistan; 219/CNBS/13, Mozambique; OOUTH/DA.326/T/1/, Nigeria).<br><br>The CLIP intervention was implemented in primarily rural areas of India (February 2014 to October 2016), Pakistan (February 2014 to December 2016), Nigeria (March 2014 to January 2016), and Mozambique (February 2015 to February 2017). Within each country, clusters consisted of an established unit of the health system (ie, primary health centre in India, union council in Pakistan, administrative post in Mozambique, and local government area in Nigeria.<br><br>In intervention clusters, community health workers were trained to provide mobile health-guided contacts, that were pregnancy hypertension-oriented, antenatal and postpartum care at home (India, Pakistan, and Mozambique) or at a primary health centre (Nigeria). Women in control clusters received usual care, advocated by the World Health Organization. In none of the study |

countries did community health workers usually either manage pregnancy hypertension or carry with them blood pressure measurement devices or antihypertensive medication.

Digital health-guided clinical assessments were recommended every 4 weeks until 28 weeks gestation, every 2 weeks from 28 to 35 weeks, weekly from 36 weeks until delivery, once within 24 hours of birth, and postnatally around postpartum days 3, 7, and 14; in Nigeria, visits were opportunistic when women attended the primary health centre.

For generalisability, as systolic blood pressure can be identified by the return of a radial pulse without the requitement for a stethoscope, the CLIP trial protocol selected a systolic blood pressure of at least 140 mmHg as the threshold at which community health workers would respond by initiating referral. In addition, community health workers were directed by the app to either administer oral methyldopa 750 mg for blood pressure of 160/110 mmHg or higher; administer intramuscular magnesium sulphate 10 g for suspected severe pre-eclampsia (miniPIERS [Pre-eclampsia Integrated Estimate of RiSk] risk for an adverse maternal outcome of at least 25%, severe systolic hypertension [at least 160 mmHg], eclampsia, stroke, or vaginal bleeding); or refer the woman to a comprehensive emergency obstetric care facility for suspected pre-eclampsia or increased risk of stillbirth (4+ dipstick proteinuria value, or absent fetal movements for at least 12 hours).[10,14]

**The PRECISE observational cohorts**

Briefly, in each participating country, 2 cohorts of women were recruited to optimise the identification of social, clinical, and biomarker determinants of the placental disorders of pregnancy, with purposeful sampling of both urban-dwelling and rural-dwelling women.[16]

First, unselected pregnant women planning to give birth in the facility (primary health centre) were approached and recruited at the time of their booking visit

| | | | | |
|---|---|---|---|---|
| | | | | for antenatal care.[16] Consenting women answered an in-depth questionnaire regarding social determinants and clinical history, underwent a targeted examination (eg, biometry, blood pressure, pulse oximetry), and provided biological samples (blood, urine, and vaginal swabs). Participants were followed-up (i) for a second antenatal research visit in the third trimester, at least 8 weeks after the first visit (questionnaire, targeted examination, and phlebotomy); (ii) the birth episode (both antepartum and postpartum; questionnaire, targeted examination, phlebotomy, vaginal swabs, placental, membrane, and cord blood samples); and (iii) 6 weeks to 6 months postpartum (with their infant, if alive; questionnaire, targeted maternal and infant examination, phlebotomy, vaginal swabs, and heel-prick samples).<br><br>Second, for comparison and to understand the lives and biology of sub-Saharan African non-pregnant women of reproductive age (16-49 years) identified as either accompanying the pregnant women described below or attending for family planning services (Kenya and Mozambique) or recruited in their homes by research staff accompanying the district health surveillance system team (The Gambia). Consenting women answered an in-depth questionnaire regarding social determinants and clinical history, underwent a targeted examination (eg, biometry, blood pressure, pulse oximetry), and provided biological samples (blood, urine, and vaginal swabs).[16] |
| Participants | 6 | (*a*) *Cohort study*—Give the eligibility criteria, and the sources and methods of selection of participants. Describe methods of follow-up<br>*Case-control study*—Give the eligibility criteria, and the sources and methods of case ascertainment and control selection. Give the | 7-8 | As above (we are treating the intervention arms of the CLIP trials as cross-sectional observational cohorts for this purpose). |

| | | | | |
|---|---|---|---|---|
| | | rationale for the choice of cases and controls<br>*Cross-sectional study*—Give the eligibility criteria, and the sources and methods of selection of participants | | |
| | | (*b*) *Cohort study*—For matched studies, give matching criteria and number of exposed and unexposed<br>*Case-control study*—For matched studies, give matching criteria and the number of controls per case | | |
| Variables | 7 | Clearly define all outcomes, exposures, predictors, potential confounders, and effect modifiers. Give diagnostic criteria, if applicable | 8 | Research staff (community health workers in CLIP and nurses in PRECISE) were trained to have women rest seated for 5 minutes, and then to measure blood pressure in a standardised fashion, with the left arm supported and the cuff at the level of the heart, at least twice. All blood pressure readings were entered manually into a study-specific digital platform, which averaged them as the blood pressure for that visit; the first and second readings were averaged unless they were more than 10 mmHg different, in which instance a third reading was requested by the study digital platform and the second and third readings were averaged.[12] |
| Data sources/ measurement | 8* | For each variable of interest, give sources of data and details of methods of assessment (measurement). Describe comparability of assessment methods if there is more than one group | 8-9 | All blood pressure readings were used for the analyses.<br><br>We determined the incremental change in systolic blood pressure and diastolic blood pressure between the first and second measurements, and the second and third measurements and first and third measurements, if a third measurement was taken. |
| Bias | 9 | Describe any efforts to address potential sources of bias | - | N/A |

| | | | | |
|---|---|---|---|---|
| Study size | 10 | Explain how the study size was arrived at | - | Opportunistic sample with nearly 1 million individual measurements. |
| Quantitative variables | 11 | Explain how quantitative variables were handled in the analyses. If applicable, describe which groupings were chosen and why | 8-9 | Individual blood pressure value frequencies were displayed for both systolic blood pressure and diastolic blood pressure for each study site, cumulatively for both the CLIP trials and the PRECISE cohorts, and cumulatively for the combined dataset. This was to identify if there was any evidence of terminal digit preference and threshold avoidance. Through that step, evidence of terminal digit preference was confirmed, over-reporting of the diastolic blood pressure value of 62 mmHg became apparent (see Results, below), and, in the CLIP dataset, suspicion of threshold avoidance at 140 mmHg systolic was observed.<br><br>We compared the directly observed counts for diastolic blood pressures of 62 mmHg vs the averaged count for diastolic blood pressures of 62 mmHg from the duplicate measurement (i.e., 1 and 2 of two measurements or 2 and 3 of three measurements) using a Fisher's exact test and Koopman asymptotic score to calculate 95% confidence intervals for the relative risk. The percentage of values ending in '0' and '5' were calculated and compared with the expected value of 10% (assuming a uniform distribution of digits) using both raw and averaged measurements using a one-sample test for proportion.<br><br>To assess threshold avoidance in the CLIP dataset (where an intervention was associated with systolic hypertension) we fit a segmented regression model to the number of blood pressure measurements per unit. Specifically, we used a Poisson model with the following covariates: blood pressure level, threshold (above and below 140 mmHg) and an interaction between these terms. We display fitted values from these models graphically and used a likelihood ratio test comparing models with and without the segmentation to assess evidence the possible discontinuity corresponding to threshold avoidance. We compared these results with those CLIP diastolic blood pressure values and |

| | | | | |
|---|---|---|---|---|
| | | | | PRECISE systolic and diastolic blood pressure values, where no relevant clinical decision thresholds were set. |
| Statistical methods | 12 | (*a*) Describe all statistical methods, including those used to control for confounding | 8-9 | **Statistical analyses**<br><br>All blood pressure readings were used for the analyses.<br><br>We determined the incremental change in systolic blood pressure and diastolic blood pressure between the first and second measurements, and the second and third measurements and first and third measurements, if a third measurement was taken.<br><br>Individual blood pressure value frequencies were displayed for both systolic blood pressure and diastolic blood pressure for each study site, cumulatively for both the CLIP trials and the PRECISE cohorts, and cumulatively for the combined dataset. This was to identify if there was any evidence of terminal digit preference and threshold avoidance. Through that step, evidence of terminal digit preference was confirmed, over-reporting of the diastolic blood pressure value of 62 mmHg became apparent (see Results, below), and, in the CLIP dataset, suspicion of threshold avoidance at 140 mmHg systolic was observed.<br><br>We compared the directly observed counts for diastolic blood pressures of 62 mmHg vs the averaged count for diastolic blood pressures of 62 mmHg from the duplicate measurement (i.e., 1 and 2 of two measurements or 2 and 3 of three measurements) using a Fisher's exact test and Koopman asymptotic score to calculate 95% confidence intervals for the relative risk. The percentage of values ending in '0' and '5' were calculated and compared with the expected value of 10% (assuming a uniform distribution of digits) using both raw and averaged measurements using a one-sample test for proportion.<br><br>To assess threshold avoidance in the CLIP dataset (where an intervention was associated with systolic hypertension) we fit a segmented regression model to |

| | | | |
|---|---|---|---|
| | | | the number of blood pressure measurements per unit. Specifically, we used a Poisson model with the following covariates: blood pressure level, threshold (above and below 140 mmHg) and an interaction between these terms. We display fitted values from these models graphically and used a likelihood ratio test comparing models with and without the segmentation to assess evidence the possible discontinuity corresponding to threshold avoidance. We compared these results with those CLIP diastolic blood pressure values and PRECISE systolic and diastolic blood pressure values, where no relevant clinical decision thresholds were set.<br><br>All analyse were performed in R statistical software version 4·0 3. P <0·01 was used for statistical significance to adjust for multiple comparisons. |
| | (*b*) Describe any methods used to examine subgroups and interactions | 9 | To assess threshold avoidance in the CLIP dataset (where an intervention was associated with systolic hypertension) we fit a segmented regression model to the number of blood pressure measurements per unit. Specifically, we used a Poisson model with the following covariates: blood pressure level, threshold (above and below 140 mmHg) and an interaction between these terms. We display fitted values from these models graphically and used a likelihood ratio test comparing models with and without the segmentation to assess evidence the possible discontinuity corresponding to threshold avoidance. We compared these results with those CLIP diastolic blood pressure values and PRECISE systolic and diastolic blood pressure values, where no relevant clinical decision thresholds were set. |
| | (*c*) Explain how missing data were addressed | - | Only complete data were used. |
| | (*d*) *Cohort study*—If applicable, explain how loss to follow-up was addressed<br>*Case-control study*—If applicable, explain how matching of cases and controls was addressed | N/A | Single values at single time points used. |

| | | | | |
|---|---|---|---|---|
| | | *Cross-sectional study*—If applicable, describe analytical methods taking account of sampling strategy | | |
| | | (*e*) Describe any sensitivity analyses | N/A | No sensitivity analyses |
| **Results** | | | | |
| Participants | 13* | (a) Report numbers of individuals at each stage of study—eg numbers potentially eligible, examined for eligibility, confirmed eligible, included in the study, completing follow-up, and analysed | 10 | Data were available for 51,875 eligible women, who were recruited from communities and primary health centres in 6 countries (The Gambia, India, Kenya, Mozambique, Nigeria, and Pakistan), with a median of 2841 [IQR 610 - 7862] women per included cohort. In total, 911,500 discrete systolic or diastolic blood pressure values were available for analysis. |
| | | (b) Give reasons for non-participation at each stage | - | N/A |
| | | (c) Consider use of a flow diagram | - | N/A |
| Descriptive data | 14* | (a) Give characteristics of study participants (eg demographic, clinical, social) and information on exposures and potential confounders | T1 | See Table 1 |
| | | (b) Indicate number of participants with missing data for each variable of interest | - | N/A |
| | | (c) *Cohort study*—Summarise follow-up time (eg, average and total amount) | - | N/A |
| Outcome data | 15* | *Cohort study*—Report numbers of outcome events or summary measures over time | | |
| | | *Case-control study*—Report numbers in each exposure category, or summary measures of exposure | | |
| | | *Cross-sectional study*—Report numbers of outcome events or summary measures | 10 | Data were available for 51,875 eligible women, who were recruited from communities and primary health centres in 6 countries (The Gambia, India, Kenya, Mozambique, Nigeria, and Pakistan), with a median of 2841 [IQR 610 - |

| | | | | |
|---|---|---|---|---|
| | | | | 7862] women per included cohort. In total, 911,500 discrete systolic or diastolic blood pressure values were available for analysis |
| Main results | 16 | (*a*) Give unadjusted estimates and, if applicable, confounder-adjusted estimates and their precision (eg, 95% confidence interval). Make clear which confounders were adjusted for and why they were included | 10 | First blood pressure measurements tended to be higher compared with subsequent measurements, but not consistently (table 2). 25,693 of 183,504 (14.0%) measurements were taken in triplicate. The raw and averaged blood pressure profiles from the CLIP trials and PRECISE Network (figure 1) examined blood pressure distributions, terminal digit preference, and the unanticipated rate of diastolic blood pressure values at 62 mmHg.<br><br>There was clear evidence of terminal digit preference for raw values ending in '0' (129,539 of 911,500 values;14·2%) and '5' (98,359 of 911,500 values; 10·8%) vs 10% expected (table 2, p < 0·001 for both '0' and '5'); these were obviated in the averaged values for '0' (39,607 of 390,697 values; 10·1%) and '5' (36,457 of 390,697 values; 9·3%).<br><br>There was a greater than expected frequency of diastolic blood pressure values of 62 mmHg (for raw observations, 28,929 of 437,446 (6·6%) vs, for averaged observations, 9310 of 195,349 (4·8%); p < 0·001; relative risk 1·094 [95% confidence interval 1·09 - 1·10], table 2, figure 1). These errors in data recording into digital platforms were largely obviated by averaging the systolic blood pressure and diastolic blood pressure values (first and second or, if required, second and third) (table 2, figure 1). The systolic blood pressure values corresponding to these 62 mmHg diastolic values varied considerably and were approximately normally distributed (figure 2); in both cohorts very few of these values were at least 140 mmHg (0·2% in CLIP, 0·7% in PRECISE) or at least 160 mmHg (0.1% in both cohorts).<br><br>For systolic blood pressure readings, there was evidence of both threshold (140 mmHg) preference (India, p = 0·02) and threshold avoidance in Mozambique (CLIP, p = 0·007; PRECISE, p = 0·006) and Pakistan (p < 0·001), but for neither The Gambia (p = 0·31), Kenya (p = 0·85), nor Nigeria (p = 0·50) (figure 3). These observations were modified by averaging values (The Gambia |

| | | | | |
|---|---|---|---|---|
| | | | | p = 0·05; India p = 0·47; Kenya p = 0·05; Mozambique CLIP p = 0·01, PRECISE p = 0·35; Nigeria p = 0·76; Pakistan p < 0·001) (figure 3). For diastolic blood pressure readings, there was evidence of threshold (90 mmHg) preference (India p <0·001; Pakistan p < 0·001) and avoidance (Mozambique CLIP p <0·001, PRECISE p = 0·008), but neither for The Gambia (p = 0·11), Kenya (p = 0·70), nor Nigeria (p = 0·05) (figure 3). Generally, these observations were modified by averaging values (The Gambia p = 0·44; India p < 0·001; Kenya p = 0·06; Mozambique CLIP p < 0·001, PRECISE p = 0·49; Nigeria p = 0·08; Pakistan p = 0·03) (figure 3). |
| | | (*b*) Report category boundaries when continuous variables were categorized | 10 | For systolic blood pressure readings, there was evidence of both threshold (140 mmHg) preference (India, p = 0·02) and threshold avoidance in Mozambique (CLIP, p = 0·007; PRECISE, p = 0·006) and Pakistan (p < 0·001), but for neither The Gambia (p = 0·31), Kenya (p = 0·85), nor Nigeria (p = 0·50) (figure 3). These observations were modified by averaging values (The Gambia p = 0·05; India p = 0·47; Kenya p = 0·05; Mozambique CLIP p = 0·01, PRECISE p = 0·35; Nigeria p = 0·76; Pakistan p < 0·001) (figure 3). For diastolic blood pressure readings, there was evidence of threshold (90 mmHg) preference (India p <0·001; Pakistan p < 0·001) and avoidance (Mozambique CLIP p <0·001, PRECISE p = 0·008), but neither for The Gambia (p = 0·11), Kenya (p = 0·70), nor Nigeria (p = 0·05) (figure 3). Generally, these observations were modified by averaging values (The Gambia p = 0·44; India p < 0·001; Kenya p = 0·06; Mozambique CLIP p < 0·001, PRECISE p = 0·49; Nigeria p = 0·08; Pakistan p = 0·03) (figure 3). |
| | | (*c*) If relevant, consider translating estimates of relative risk into absolute risk for a meaningful time period | - | N/A |
| Other analyses | 17 | Report other analyses done—eg analyses of subgroups and interactions, and sensitivity analyses | - | N/A |

| | | | | |
|---|---|---|---|---|
| **Discussion** | | | | |
| Key results | 18 | Summarise key results with reference to study objectives | 11 | Using blood pressure data from 51,875 prospectively-recruited eligible women living in 5 low- and middle-income countries, 911,500 discrete systolic or diastolic blood pressure values were analysed in this study. Our protocol of repeating (up to 3 times) and averaging blood pressure measurements (values 1 and 2 of 2, or 2 and 3 of 3) clearly improves the quality of data and clinical guidance that are derived from use of these two devices. This was true for the terminal digit preference and the algorithmic issue that resulted in the excess of diastolic blood pressure values of 62 mmHg, but not for threshold avoidance. |
| Limitations | 19 | Discuss limitations of the study, taking into account sources of potential bias or imprecision. Discuss both direction and magnitude of any potential bias | 11 | Our limitations include the absence of a Bluetoothed device for comparison, by which means we could test the hypothesis that the terminal digit preference and threshold avoidance could be overcome by Bluetoothed data collection onto digital health platforms. |
| Interpretation | 20 | Give a cautious overall interpretation of results considering objectives, limitations, multiplicity of analyses, results from similar studies, and other relevant evidence | 12 | In summary, the BP 3AS1-2 and CRADLE VSA have been used reliably in a number of other clinical trials and studies with largely positive results.[18-21] They have provided generally reliable, low cost blood pressure monitors that can be safely deployed from households to facilities in low- and middle-income countries. The addition of the traffic lights in the CRADLE VSA appears to have reduced the risk of threshold avoidance. However, in this study, we have identified opportunities for further improvements that relate to terminal digit preference and the algorithm issues around 62 mmHg. In the interim, colleagues should use our protocol for using these devices 2-to-3 times to reduce errors. |
| Generalisability | 21 | Discuss the generalisability (external validity) of the study results | 11 | Reassuringly for clinical use of these two devices, 62 mmHg diastolic readings were rarely (less than or equal to 0·7%) associated with a systolic blood pressure value of at least 140 mmHg and only 0·1% were associated with systolic readings of at least 160 mmHg. In addition, it might be useful to assess whether or not the 62 mmHg issue is shared across Microlife automated and semi-automated blood pressure devices. |

Table 14

| | | | | |
|---|---|---|---|---|
| | | | | Within the CLIP trials, the addition of the traffic light display may underlie why there was no evidence of threshold avoidance in the CLIP Nigeria pilot trial. The CLIP Nigeria pilot trial was the sole part of the CLIP endeavour that used the then new CRADLE VSA device,[9] compared with the BP 3AS1-2 device used in India (threshold preference), and Mozambique and Pakistan (threshold avoidance) were clearly seen for systolic, but less so diastolic, hypertension, as the protocol was systolic blood pressure-driven.[6-8,10] The display of an amber or red light that was visible to others in the room when the blood pressure was taken may have provided important shared information to guide shared decision-making about initiating transfers of care and, in some cases, methyldopa and magnesium sulphate therapy. However, both systolic threshold preference and threshold avoidance was observed in the Gambian and Mozambican, but not Kenyan, PRECISE cohort. Diastolic preference and avoidance were both seen, but were of lower magnitude than for systolic blood pressure values. It would be interesting to understand why Gambian research staff and Indian ASHA workers preferred a value diagnostic of hypertension, while Mozambican research staff and APEs and Pakistani LHWs displayed threshold avoidance. Were there differences in the training about the importance of detecting hypertension, or were there concerns about the additional workload associated with making such a diagnosis? |
| **Other information** | | | | |
| Funding | 22 | Give the source of funding and the role of the funders for the present study and, if applicable, for the original study on which the present article is based | 3-4 | UK Research and Innovation Global Challenges Research Fund (through the Medical Research Council) and the University of British Columbia, a grantee of the Bill & Melinda Gates Foundation. |

*Give information separately for cases and controls in case-control studies and, if applicable, for exposed and unexposed groups in cohort and cross-sectional studies.

**Note:** An Explanation and Elaboration article discusses each checklist item and gives methodological background and published examples of transparent reporting. The STROBE checklist is best used in conjunction with this article (freely available on the Web sites of PLoS Medicine at

http://www.plosmedicine.org/, Annals of Internal Medicine at http://www.annals.org/, and Epidemiology at http://www.epidem.com/). Information on the STROBE Initiative is available at www.strobe-statement.org.
